# Effects of Replacing Groundnut Cake with Blood Vegetable Waste Meal in the Diets of Weaner Rabbits

**DOI:** 10.5402/2012/321516

**Published:** 2012-01-23

**Authors:** Adedayo Abiodun Adeniji

**Affiliations:** Department of Animal Science, Faculty of Agriculture, University of Abuja, P.M.B. 117, Abuja, Nigeria

## Abstract

A total of seventy-two weaner rabbits of eight weeks of age were used to assess the effects of replacing groundnut cake (GNC) with blood vegetable waste meal (BVWM) in the diets of rabbits. The BVWM was fed to replace dietary GNC at 0, 15, 30, and 45%, with GNC being 15% in the control diet. The four experimental diets were fed *ad libitum* for a period of eight weeks. BVWM was analyzed to contain a crude protein value of 62.35%. There were comparable feed intake values by rabbits on all the diets although the rabbits in the higher replacement levels of BVWM tended to have consumed more of the feed. There were significant increases (*P* < 0.05) in body weight gain by rabbits as the BVWM level increased in the diet. Similarly, the feed to gain ratio improved and nitrogen digestibility increased (*P* < 0.05) with higher levels of BVWM in the diet. This study shows that rabbits can tolerate the 45% BVWM replacement of groundnut cake effectively.

## 1. Introduction

Discarded* Amaranthus cruentus *is a form of vegetable that cannot be consumed by humans and are ready for disposal after 48 hrs of being harvested. *Amaranthus cruentus* is species of vegetable that is produced abundantly in most African counties. It serves as spices in human diets [1] but begins to deteriorate after 48 hrs of harvest. When unsold and discarded, it becomes a total loss to the seller.

Conventional protein feedstuffs such as soyabean meal, ground nut cake, and fish meal are quite expensive. The high cost of these ingredients has resulted in the inadequate/poor feeding of rabbits and other livestock species. The need to sources for alterative feedstuffs so as to reduce the high cost of livestock production cannot be overemphasized. Blood vegetable waste meal (BVWM) is obtained from processed blood and vegetable; both are waste. The blood is washed away at the abattoir, while the unsold vegetable is discarded at refuse site. Hence, BVWM is obtained free and can be fed to rabbits to reduce production cost, particularly as feed accounts for almost 70% of the cost of production.

Rabbit has come under focus as an animal because of its several potentials. It has small body size, short gestation interval, induced ovulator, and an ability to utilize forage. Rabbits do not really compete with humans in terms of feeding. This is because they can feed on vegetative-based diets and kitchen waste. Rabbits are hindgut fermenters, capable of utilizing poor quality feed, and can tolerate up to 25% dietary crude fibre [2].

Improved rabbit production can play important role in increasing animal protein supply to under nourished populace, considering the high cost of chicken, pork, and beef. The study was aimed at investigating the effects of replacing groundnut cake with BVWM on the performance of rabbits and to also ascertain what level of BVWM that can best replace groundnut cake (GNC).

## 2. Materials and Methods

Discarded *Amaranthus cruentus* (which was not sold 48 hours after harvest; they are considered as waste and are about to be thrown away) was collected from the market and processed along with fresh bovine blood at ratio 1 : 2 (w/w), that is, one of blood to two of vegetable. The discarded vegetable and blood were thoroughly mixed and cooked at 100°C for about 1.5 hours. It was stirred regularly to prevent burning. The cooked mixture blood vegetable waste meal (BVWM) was later sun-dried until the moisture content was below 15%. On analysis, BVWM contained 62.35% crude protein, 6.4% crude fibre, and 3.45% ether extract.

A total of 72 eight-week old weaner rabbits of both sexes were used in this study there were four treatments, with each treatment having 3 replicate groups, and there were 6 rabbits housed individually placed in each replicate. There are four experimental diets (Table 1) which are such that BVWM was fed to replace dietary groundnut cake (GNC) at 0, 15, 30, and 45%, while GNC in the control was 15%.

All rabbits were individually housed in metal cages throughout the feeding trial which lasted for a period of eight (8) weeks. Feed and water were supplied *ad libitum* to the experimental animal.

Initial and weekly body weight records and daily feed intake record were kept. The feed to gain ratio was calculated. At the last week of feeding trial, faecal and urine samples were collected for 3 consecutive days by the total collection method to determine nitrogen digestibility. All proximate analysis were conducted by the method of AOAC [3]. Data collected were subjected to statistical analysis appropriate for the completely randomized design [4].

## 3. Results

Results of growth performance of rabbits fed BVW are presented in Table 2. There was an increase in body weight gain by rabbits as the level of replacement with BVWM increased in their diet. The rabbits on the control diet had a body weight gain of 7.28 g/day which was comparable (*P* > 0.05) to the body weight gained by rabbit on the 15%. BVWM replacement diet (7.95 g); but significantly lower (*P* < 0.05) to the body weight gain values for the rabbits on 30 and 45% BVWM replacement diets that gained 9.69 and 9.38 g, respectively. The feed intake values were comparable (*P* > 0.05) for rabbits on all the diets, but these feed intake values tended to increase as the BVWM replacement level increased in the diets.

The feed to gain ratio was significantly (*P* < 0.05) affected by the treatment. These ratios decreased as the BVWM replacement level increased in their feed. The rabbits on the 30 and 45% BVWM replacement diet had significantly lower (*P* < 0.05) feed to gain ratio values (6.89 and 6.80, resp.) compared with the 0 and 15% BVWM diets that had feed to gain ratios of 7.85 and 7.81, respectively.

Table 3 shows the results of nitrogen digestibility of rabbits when groundnut cake was replaced with BVWM. Although nitrogen intake was comparable (*P* > 0.05) for rabbits on all the diets, the intake seemed to have increased with the increasing levels of BVWM replacement in the fed. Faecal nitrogen values were highly comparable (*P* > 0.05) for all treatments except rabbits fed on the 45% BVWM replacement diet that had significantly lower (*P* < 0.05) faecal nitrogen value of 0.37 g.

The rabbits had comparable (*P* > 0.05) nitrogen balance values except for the rabbits on the 45% BVWM replacement diet with a higher (*P* < 0.05) nitrogen balance of 1.76 g. Nitrogen digestibility increased (*P* < 0.05) as the level of BVWM replacement also increased in the rabbit diet. The rabbits on the control feed had a digestibility of 60.49% which kept increasing up to 76.86% for the rabbits fed 45% BVWM diet.

## 4. Discussion

The rabbits seemed to have much appetite for the test ingredient (BVWM). The rabbits tended to consume more of the BVWM diets than the groundnut cake control diets. This is contrary to reports of Adeniji [5], who reported decreased feed intake by broiler finishers as the BVWM diets increased. Rabbits as nonruminant herbivores can tolerate more of fibrous feeds than poultry; hence, the better feed consumption was obtained in this study. The increased body weight gain obtained in the study with the higher levels of BVWM in the rabbit feed implies that BVWM possessed a growth stimulating effect. BVWM is high in protein (62.35%). The protein value is comparable with that of local fishmeal (65%). Vegetable diets have increased growth rates. Similarly, Samkol et al., [6], Tam et al. [7], and Dong et al. [8] reported improved growth rate when water spinach was fed to rabbits.

The improved feed to gain ratio at the high levels of BVWM shows that BVWM is a promising feedstuff. BVWM can help to solve the problem of feed scarcity and reduce demand on expensive protein supplements like groundnut cake and soyabean meal. Akinfala et al. [9] and Ekenyem and Madubuike [10] reported that there are lots of tropical foliages that can be fed as based diets for rabbits.

The improved digestibility with higher levels of BVWM implies that rabbits are able to effectively digest higher levels of BVWM whose could have been discarded as waste. BVWM which monogastrics might not be able to digest well because of their high fibre level should not be discarded but converted for rabbit feed.

Based on the lower feed to gain ratio and higher nitrogen digestibility, rabbits can tolerate the 45% groundnut cake replacement with BVWM. This will reduce the heavy demand for groundnut cake in livestock feeding and its high price reduced. Improved rabbit production can play on important role in increasing animal protein supply to people in the under developed countries, considering the high cost of chicken, pork, and beef.

## Figures and Tables

**Table 1:** Composition of experimental diets (Kg/100 kg).

Ingredients	0%	15%	30%	45%
Maize	42.50	42.50	42.50	42.50
Palm kernel cake	1150	11.50	11.50	11.50
Wheat offal	16.00	16.00	16.00	16.00
Groundnut cake	15.00	15.00	15.00	15.00
BVWM	0.00	2.25	4.50	6.75
Fishmeal (72%)	2.00	2.00	2.00	2.00
Maize offal	7.80	7.80	7.80	7.80
Bone meal	3.00	3.00	3.00	3.00
Limestone	1.50	1.50	1.50	1.50
Salt	0.35	0.35	0.35	0.35
*Premix	0.25	0.25	0.25	0.25
Methionine	0.10	0.10	0.10	0.10

Total	100	100	100	100

Analyzed proximate values				
Crude protein (%)	14.70	14.81	14.95	15.11
Crude fibre	7.13	7.45	7.52	3.66
Ether extract	3.44	3.56	3.50	3.66

*The premix used was produced by Animal Care Services Konsult (Nig.) Ltd., and each 2.5 kg contained vit. A: 1,000,000 iu; vit D3: 2,000,000 iu; E: 100,000 iu; vit K: 2,000 mg; thiamine: 1,500 mg; pantohenic acid: 40 mg; vit Bi2: 10 mg; folic acid: 500 mg; biotin 20 mg; c

Cu: 5 g; iodine: 12 g; selenium: 200 mg; Co: 200 mg.

**Table 2:** Growth performance of rabbits fed BVWM.

	Level of GNC replacement with BVWM				
Parameters	0	15	30	45	SEM
Initial body weight (g/Rabbit)	432.5	407.5	420.0	410.0	
Final body weight (g/Rabbit)	840.0	852.5	962.5	935.5	
Feed intake (g/Rabbit/day)	57.15	62.09	66.81	63.81	1.6 NS
Body weight (g/Rabbit/day)	7.28^b^	7.95^b^	9.69^a^	9.38^a^	4.51
Feed to gain ratio	7.85^a^	7.81^a^	6.89^b^	6.80^b^	1.61

Means in the same row followed by different superscript letter are significantly different (*P* < 0.05).

**Table 3:** Effects of GNC replacement with BVWM on nitrogen digestibility of rabbits.

	Level of GNC replacement with BVWM				
Parameters	0	15	30	45	SEM
Nitrogen intake (g)	2.05	2.14	2.21	2.29	0.1 NS
Faecal nitrogen (g)	0.58^a^	0.55^a^	0.51^a^	0.37^b^	0.02
Urinary nitrogen (g)	0.23	0.20	0.18	0.16	0.02 NS
Total nitrogen output (g)	0.18	0.75	0.69	0.53	
Nitrogen balance (g)	1.24^b^	1.39^b^	1.52^ab^	1.76^a^	0.1
Nitrogen digestibility (%)	60.49^b^	64.95^b^	68.78^ab^	76.86^a^	2.0

Means in the same row followed by different superscript letter are significantly different (*P* < 0.05).
